# Prevalence of flatfoot in school between 3 and 10 years. Study of two different populations geographically and socially

**Published:** 2012-06-30

**Authors:** Enrique Vergara-Amador, Rafael Fernando Serrano Sánchez, Juan Rafael Correa Posada, Adriana Carolina Molano, Oscar A Guevara

**Affiliations:** Unidad De Ortopedia Departamento De Cirugía Universidad Nacional De Colombia Profesor asociado de ortopedia; Unidad De Ortopedia Departamento De Cirugía Universidad Nacional De Colombia Ortopedista; Unidad De Ortopedia Departamento De Cirugía Universidad Nacional De Colombia Ortopedista; Unidad De Ortopedia Departamento De Cirugía Universidad Nacional De Colombia Medico Cirujano; Unidad De Ortopedia Departamento De Cirugía Universidad Nacional De Colombia Profesor Asociado de Cirugía

**Keywords:** Flatfoot, prevalence, children, school

## Abstract

**Introduction:**

Children present with flatfoot from birth and it resolves along infancy. There have been several risk factors identified for the development of flatfoot: male sex, young age, overweight and obesity. The prevalence of flatfoot decreases with age. The aim of this study was to determine the prevalence of flatfoot in two different populations with different social, cultural and geographically characteristics in Colombia.

**Methods:**

This is a cross sectional study made on school children between 3 to 10 years of age, from several schools in Bogota and Barranquilla. From 940 total children 60% were from Bogota. Flatfoot was diagnosed according to physical exam.

**Results:**

We found a global prevalence of flatfoot of 15.7%, distributed 20.8% in Bogota and 7.9% in Barranquilla. The children from 3 to 5 years had a prevalence of 30.9%, decreasing significantly after this age. It was found that children 3 to 5 years old from Bogota had a prevalence of flatfoot of 38.3% while children from Barranquilla only 17.3%, decreasing significantly in children older than 6 years. In the multivariate analysis we found an association between flatfoot with age, city, gender and body mass index.

**Discussion:**

We found a bigger prevalence of flatfoot in the population of Bogota compared to Barranquilla suggesting an influence of social, cultural and racial factors in the development of flatfoot. The diminished prevalence of flatfoot in children over 6 years of age suggest that therapeutic measures before this age are not recommended.

## Abstract (es)

**Introducción:**

Los niños presentan el arco plantar plano al nacimiento, el cual se desarrolla durante la primera década de la vida. Han sido identificados como factores de riesgo para pie plano: el sexo masculino, menor edad, el sobrepeso y la obesidad. La prevalencia del pie plano disminuye con la edad. El objetivo general del estudio es determinar la prevalencia del pie plano en dos poblaciones diferentes desde el punto de vista geográfico, cultural y social en Colombia.

**Métodos:**

Es un estudio de prevalencia, de corte transversal, realizado en niños entre 3 y 10 años, estudiantes de colegios de Bogotá y Barranquilla. Se evaluaron 940 niños, 60% en la ciudad de Bogotá. Se determinó el pie plano mediante el examen físico

**Resultados:**

Se encontró una prevalencia global de pie plano de 15.7%, en Bogotá de 20.8% y en Barranquilla 7.9%. Los niños en el rango de 3 a 5 años tuvieron una prevalencia de 30.9%, disminuyendo de forma significativa después de esta edad. Se encontró que los niños de Bogotá entre 3 y 5 años tenían una prevalencia de pie plano de 38.3 % y los de Barranquilla tan solo 17.3 %, disminuyendo significativamente en los mayores de 6 años. En el análisis multivariable se encontró asociado el pie plano con la edad, ciudad, género y el índice de masa corporal.

**Discusión:**

Se tuvo una mayor prevalencia en Bogotá comparada con Barranquilla en las poblaciones estudiadas sugiriendo la influencia de factores culturales, sociales y raciales en la aparición de pie plano. El descenso importante en la prevalencia en los mayores de 6 años, sugiere limitar el uso de medidas terapéuticas antes de esta edad.

## Introduction

Pesplanus (flat foot) is one of the most frequent causes of orthopedic consultation, with the development of the foot arches initiating during the first decade of life^1^ ^-^ ^2^. The natural history of flat-footedness is unknown, although some studies suggest that the majority of flat feet are asymptomatic during adulthood^2^. Flat-footedness may exist as an isolated condition or it may be associated to a broader clinical condition that may include ligament laxity, muscular or neurological anomalies, genetic conditions, and collagen disorders^3^.

Flat-footedness may be classified as pathological or physiological. The pathological, or rigid flatfoot, is characterized by a fixed arch that is not modified by the support or lack of support of weight^3^. It has multiple etiologies and leads to pain and disability, often requiring treatment given that it is associated to an underlying pathology^1^ like congenital vertical talus, tarsal bars, idiopathic short Achilles tendon, and accessory scaphoid bone. 

The physiological or flexible flatfoot, characterized by a normal arch without weight support and flattening of the arch during standing^3^, is often noted during the first decade of life and can be symptomatic or asymptomatic. Factors like ligament laxity and overweight contribute to its persistence^1^. The following have been identified as risk factors for flat-footedness: male gender, lower age, overweight, and obesity^1^.

The prevalence for flat-footedness diminishes significantly with age.In three-year old patients, the condition has been reported up to 54% and in the six-year-old group it has been reported at 24%^1^. Garcia *et al*.^4^, found diminished prevalence of flat feet in children from low and low-middle class families, with a predominance of the diagnosis in males over females in both social classes^4^. Most children show full development of normal feet by 12 years of age^4^.

In normal feet, 61% of the weight is supported in the posterior area, 35% in the anterior area, and only 4% in the mid zone. In flat feet, between 17 and 30% is supported by the mid zone^2^. The load changes from the lateral column to the medial column, which leads to an abnormal gait in patients with flat feet. It is felt that flat feet in obese children may be caused by the presence of a plantar fatty pad under the medial longitudinal arch of the foot^5^, which diminishes between two and five years of age when the arch is formed. Another cause suggested for flat-footedness in obese children is that it can be caused by the collapse of the medial longitudinal arch because of excessive and continuous load on the foot by the body mass. A study by Mickle *et al*., showed that flat-footedness is more common in overweight and obese children than in children with normal weight, as a consequence of changes in the structure of the foot, especially of the medial longitudinal arch^5^.

Some authors found relationship between wearing shoes at an early age and flatfoot. Genetic factors and age determine the shape of the foot's arch^6^. The clinical diagnosis of flatfoot is based on the valgus position of the heel and the non-formation of a medial arch^1^.

During preschool age, children with flexible flat feet have a valgus < 20° and active correction is possible. Pathological flat-footedness is defined by a valgus >20° and the impossibility for active correction of the heel valgus^1^. 

 The critical age for developing the arch is six years of age, thereby, if the prevalence of this pathology is studied in children younger than six years of age, the diagnosis will be overestimated^4^.

The general objective, herein, was to determine the prevalence of flat feet in two different populations from a geographic, cultural, and social point of view in Colombia, describe the population studied, and try to determine the association of flat feet with risk factors and with symptoms present.

## Materials and Methods

This was a prevalence, cross-sectional study of children between 3 and 10 years of age, studying at schools in two different cities: Bogotá, the capital of Colombia, at 2,600 m above sea level with a mean temperature of 12 °C and differences in garment and shoe wear with respect to the other city selected, which is Barranquilla, a Colombian city on the Caribbean coast with warm climate and a mean temperature of 35 °C. Inclusion criteria were: age between 3 and 10 years, students in Barranquilla or Bogotá in selected schools and having informed consent from parents or legal guardians.

Exclusion criteria included those with congenital or acquired neurological disease, prior pathology of the foot or lower limb affecting gait or support, children prior foot surgery, parents who refused to participate in the study and who did not sign the informed consent, and children who did not attend school on the day of the clinical evaluation.

The sample was taken from six school institutions from low and middle social classes; three from the city of Bogotá and three from Barranquilla. Sample size was calculated with the Stat Calc tool from EpiInfo 3.3.2, for a significance level of 0.05, power of 80%, expected frequency in Barranquilla: 12.5%, expected frequency in Bogotá: 20% Ratio 2:1 Bogotá: Barranquilla. With this, the sample size was of 310 children in Barranquilla and 620 children in Bogotá.

The following variables were considered: age, gender, height, weight, body mass index, race, city, dominance, family antecedents of foot pathology, Pfeiffer rearfoot angle^1^ (defined by the angle formed by the upper part of the Achilles tendon with the direction of the distal insertion on the calcaneus), Denis classification^4^, Jack test^6^, and Root test.

The evaluation of the clinical data and the physical exam was performed by two independent observers to avoid the subjective bias of a sole observer. A podoscope was used to assess the presence of flat feet and classify it according to Denis, along with a goniometer with which the angles described were determined, measuring them in degrees. The distance from the inner border of the foot to the support zone of the arch region was not measured.

The information was stored in a data base in Excel® 2007 (Microsoft®, Richmond, VA) and it was analyzed with the STATA(r): Release 10 statistical package (StataCorp®. 2007 College Station, TX). To describe qualitative variables, we used proportions; for quantitative variables we used means and standard deviation if they had a normal distribution or median and interquartile range if they did not have a normal distribution. Flat-footedness was classified according to the Denis classification, and children were determined as flat-footed if they had at least one type 1 in one of their feet. Finally, a multivariate analysis was performed with the regression method, seeking association of flat feet with the variables studied.

The ethical implications of this study were minimal, given that they corresponded to a study without risks, where the information provided by the parents was used without any intervention on the patients. The information obtained remained absolutely confidential and was used exclusively by the researchers.

This study was approved by the ethics committee at Universidad Nacional de Colombia.

## Results

A total of 940 children were evaluated in two cities; 573 in Bogotá (60.96%) and 367 in Barranquilla (39.04%). 

Table 1 shows the characteristics of the population studied, noting that the populations are relatively similar, except for racial characteristics.

**Table 1:**
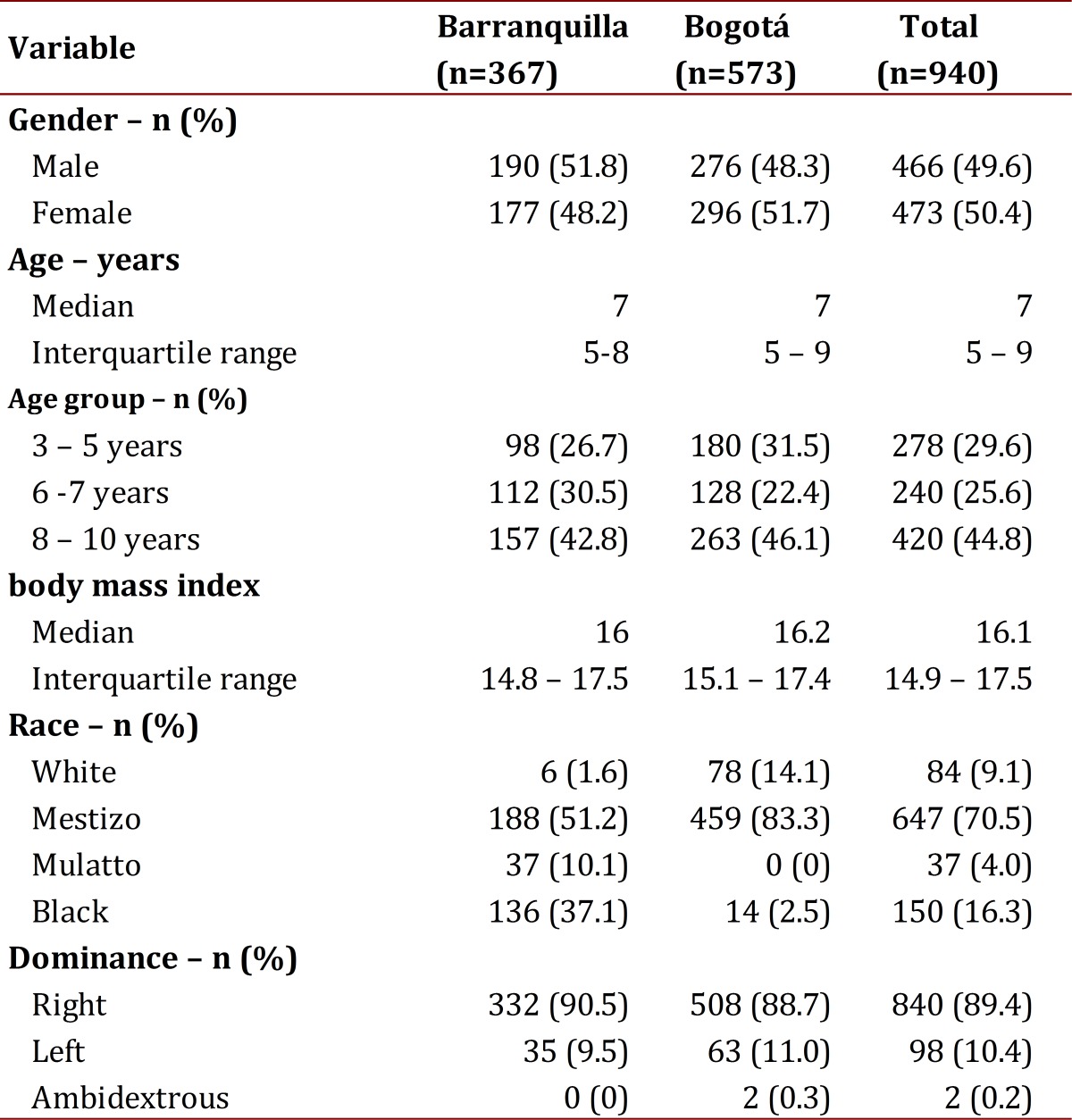
Characteristic of the populations

Table 2 reveals the results by comparing the cities of Bogotá and Barranquilla, showing greater prevalence in Bogotá. A general prevalence for flat feet was found in the study population at 15.74%.

**Table 2.**
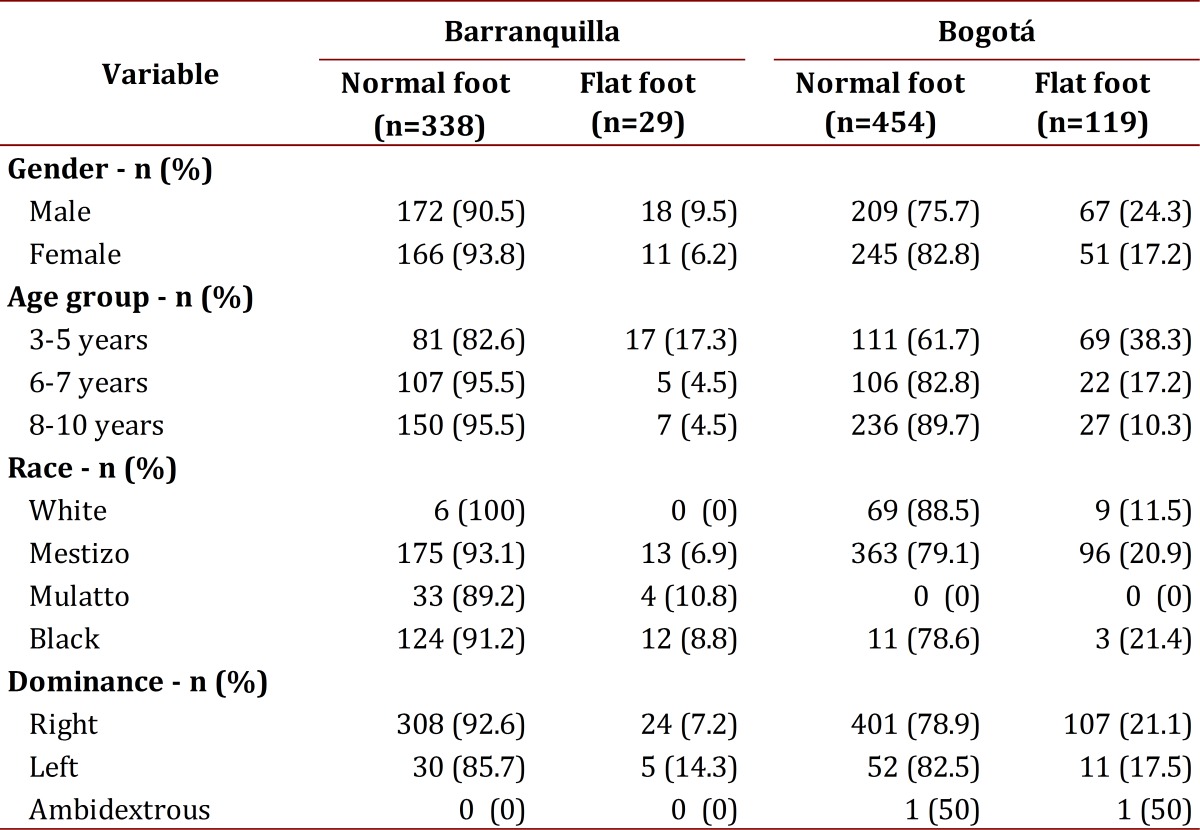
Results by city for flatfoot and normal foot

Upon analyzing the presence of flat-footedness per city, Bogotá registered a prevalence of 20.8% and Barranquilla of 7.9%. If we observe the age groups, we find in the group from 3 to 5 years of age prevalence for flat feet of 38.3% in Bogotá and 17.2% in Barranquilla, and for those older than 6 years of age of 27.5% in Bogotá and 9% in Barranquilla. Regarding gender, males had greater prevalence for flat feet at 18.2%.

Table 3 shows the findings of the Denis classification per age group with significant differences between the 3- to 5-year-old group and the older children, and there was only one patient with flatfoot Denis^3^.

**Table 3:**
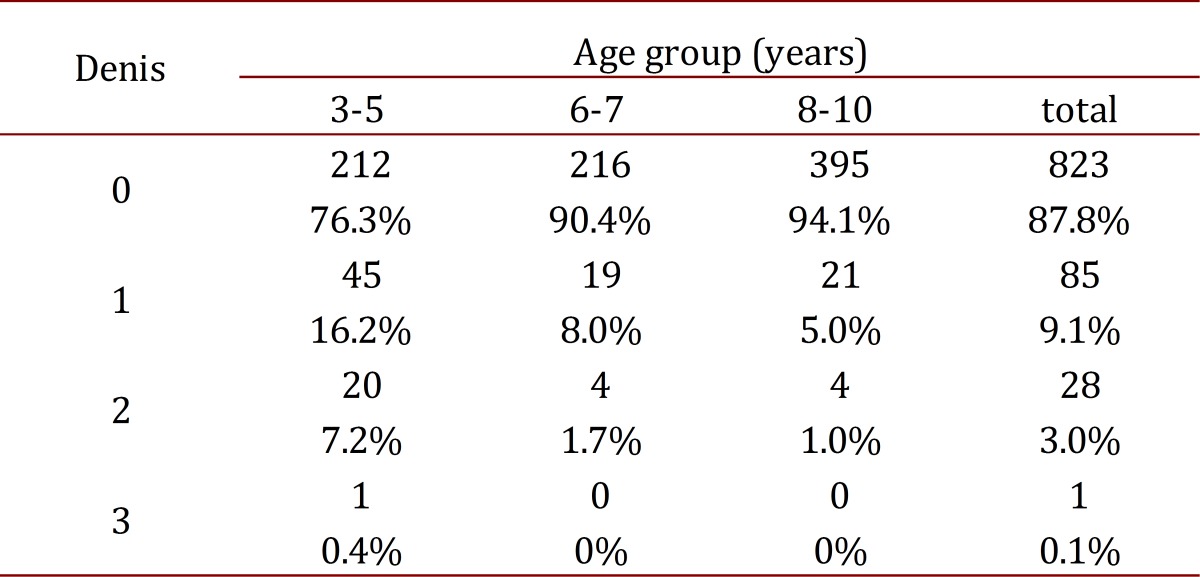
Results for denis classifications and age group

Upon performing the logistic regression, seeking the association of the different variables with flat feet, we found the results described in Table 4. The multivariate analysis found as associate factors for the presence of flat feet: the city of Bogotá, male gender, and increasing body mass index. Likewise, increased age indicated lower prevalence of flat feet.

**Table 4:**
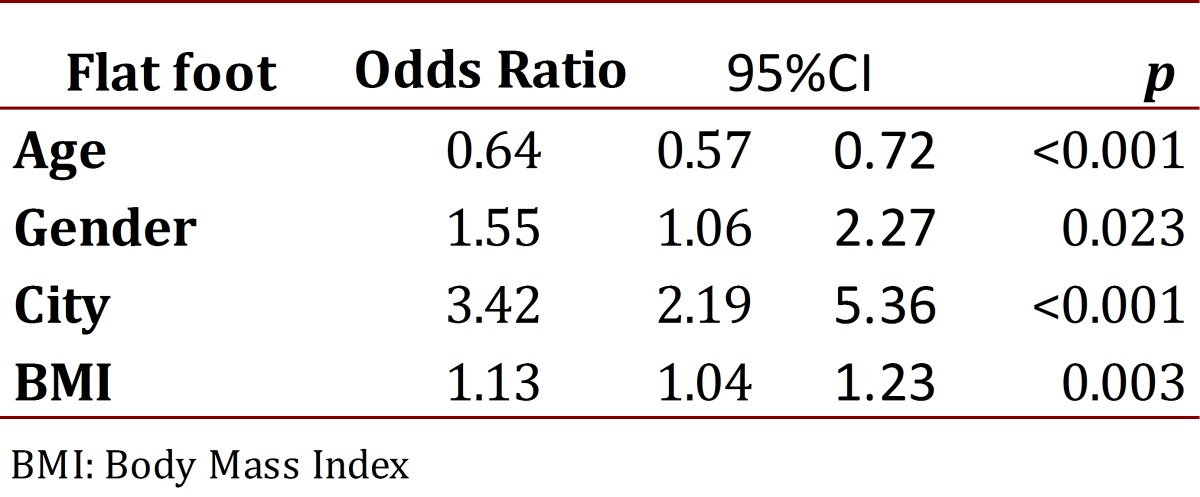
Multivariate analysis with regression method IMC: body mass index

We should note that throughout the sample examined initially, we only found one child with rigid flatfoot with antecedent of surgical correction of clubfoot who was not included in the study and who is not part of the 940 children. All the children catalogued as flatfoot were asymptomatic.

## Discussion

This study found a global prevalence of 15.74% for the population analyzed, compared to other studies ranging between 2.7 and 44% in the Pfeiffer study^1^ ^,^ ^4^. 

The difference of our results with the 2.7% prevalence reported by Garcia *et al*.^4^, seems explainable given that the study by this author considered a population that included older age groups (13 to 15 years of age) in which the arch was completely developed, considering as flat footed only those subjects with at least a Denis grade^2^.

Few studies in literature determine the prevalence of flat-footedness; among them, there is the study by Echarri and Forriol^7^ who reported 70% prevalence for flat feet in children 3 to 4 years of age and of 40% between 5 and 8 years of age. 

Lin *et al*.^8^, found 57% prevalence at 2 to 3 years of age, diminishing to 21% between 5 and 6 years of age. Arizmendi *et al*.^9^, in a Mexican population in Morelia, reported 31.9% prevalence of flat feet in preschool children and 8.8% in school-age children.was found for 2.7% of the population studied comprising children between 4 and 13 years of age^4^.

In a study conducted in the province of Málaga, Spain, prevalence.This study included populations from two different cities, Bogotá the capital of Colombia - at 2,600 m above sea level with a mean temperature of 12 °C and with cultural differences regarding garments and closed footwear and its use for longer periods of time with respect to the city of Barranquilla, a city on the Caribbean sea, where the use of open footwear like sandals is more frequent and the use of footwear is lower. We found a global prevalence of 30.9% in the age group from 3 to 5 years - 38.3% for the city of Bogotá and 17.3% for the city of Barranquilla, suggesting the influence of footwear use and its form for the development of the medial longitudinal arch.

Rao and Joseph *et al*.^10^, reported a higher prevalence of flat feet in children using enclosed footwear (8.6%) with respect to children who predominately wear sandals or do not wear shoes (2.8%). The study by Sachithanandam and Joseph^11^ also suggests an association between time of footwear use and flat foot presence.

The study by Echarri and Forriol in children from Congo used Clarke's angle and the Chippaux Smirak index to diagnose flat feet. Said study revealed that development of the foot's medial arch is influenced by three factors: age, gender, and use of footwear.

Clarke's angle is the best way of showing the influence of the use of footwear on the morphology of the flat foot, while the Chippaux Smirak index and the Stahely arch index are more convenient to detect the importance of gender in the formation of the foot arch. The three parameters found indicate the importance of age in the development of the foot print^7^.

Stahely *et al*.^12^, found statistically significant difference between males and females, but which was not important in practice, indicating that only 2% variation in the arch could be explained by the gender difference.

Also, by using Stahely's index, which is the division of the width of the foot at the median part of the foot in the arch, over the width of the heel, where the normal values are between 0.75 and 1.35; some type 1 flat feet cases, according to the Denis classification, could be considered normal.

In any event, this study preferred the Denis classification, which we deemed more practical, used in other studies, and which we have been using for years in our orthopedic service. 

Flat foot prevalence varies considerably with age, finding that 6 years of age is the age limit for the disappearance of flat foot, which is also evident in our study where children older than 6 years of age presented much lower prevalence than younger school children, similar to that reported by Arizmendi *et al*^9^.

This phenomenon has been explained through several factors like ligament laxity, overweight, and the fatty tissue in the arch, which in the group older than 6 years of age may explain the decreased prevalence by the diminished fatty tissue package and the definite conformation of the foot arch.

The study by Rao and Joseph^10^ reported as another factor related to the presence of flat foot the increase in body mass index, as also reported by Pfeiffer who even stated a three times greater risk for flat foot in overweight patients^1^.

This work also shows a greater ratio of flat feet with the male gender, similar to that reported in other studies^1^.

The prevalence reported for the population of Bogotá is significantly greater with respect to the population studied in Barranquilla and it remains so when analyzing by age groups, suggesting the presence of racial, cultural, and social conditions that would affect the persistence of flat feet.

A limitation in the current study is that because it is a cross-sectional design, the associations found must be evaluated by cohort-type studies, to better approach the causality of the problem studied.

## Conclusions

The data show significant differences in the prevalence of flat feet in the populations studied. This agrees with international reports that considered male gender and overweight as risk factors the presence of this condition. 

Definitely, the age limit in the formation of the foot is found past 6 years of age when the prevalence in all the populations reported in the literature and in our population diminishes sharply.

In this work, we found prevalence for flat foot of 30.9% in the group from 3 to 5 years of age and of 11.3% in the group from 6 to 7 years of age, suggesting that treatment with orthoses like shoe inserts or orthopedic shoes would not be indicated before 6 years of age, given that the natural course of the condition is toward improvement.

We feel that the differences found in this study for the prevalence in both populations studied may be due to racial and socio-cultural conditions like the type and use of footwear.
